# Molecular genotyping of multi-system rare blood types in foreign blood donors based on DNA sequencing and its clinical significance

**DOI:** 10.1515/med-2025-1234

**Published:** 2025-07-17

**Authors:** Jianli Gong, Xianguo Xu, Jianrong Zhu

**Affiliations:** Comprehensive Office, Yiwu Central Blood Station, Yiwu, Zhejiang, 322000, China; ZheJiang Blood Center, Institute of Transfusion Medicine, Hangzhou, Zhejiang, 310052, China; Station Master Room, Yiwu Central Blood Station, 428 Xicheng Road, Yiwu, Zhejiang, 322000, China

**Keywords:** foreign blood donors, rare blood types, DNA sequencing, genetic polymorphism, clinical transfusion, red cell survival rate

## Abstract

**Objective:**

To establish a multi-level blood type identification system, comprehensively analyze the distribution characteristics and genetic polymorphisms of multi-system rare blood types in foreign blood donors, explore the application value of DNA sequencing technology in rare blood type screening, and evaluate its clinical significance in complex transfusion patients.

**Methods:**

Blood samples from 277 foreign blood donors who participated in voluntary blood donation in Yiwu City were prospectively collected from June 2021 to March 2023. Serological typing of 24 antigens from 11 red blood cell blood group systems (ABO, Rh, Duffy, MNS, Kidd, Kell, Lutheran, P1PK, Lewis, H, and Diego) was performed using microcolumn agglutination and tube methods. First-generation sequencing technology was used to perform whole-exome sequencing of Duffy, Kell, Ss/GYPB, and Diego genes on screened rare phenotype samples to analyze genetic polymorphism characteristics. Key mutation sites were verified using multiplex PCR-sequencing. A rare blood type DNA database was established and compared with the international blood group gene database (BGMUT). Confirmed rare blood type units were preserved through programmed freezing, and their clinical application effects were tracked and analyzed.

**Results:**

The 277 foreign blood donors were primarily from the Middle East and South Asia (71.8%), with major source countries including Syria (56 cases, 20.22%), Yemen (49 cases, 17.69%), Pakistan (24 cases, 8.66%), Iraq (20 cases, 7.22%), India (15 cases, 5.42%), Iran (14 cases, 5.05%), Mali (11 cases, 3.97%), and Jordan (10 cases, 3.61%). In blood type distribution, Fya antigen expression was highest among Indian (100%) and Pakistani (87.50%) donors; 63 cases of Fy(a−b−) were found, most commonly in donors from Mali and Yemen. S antigen expression was highest in donors from Syria (60.71%), India (60.00%), and Pakistan (58.33%); 47 cases of S+s− were detected. Additionally, 12 cases of Lua+ were found, distributed among Syria (3 cases), Iraq (2 cases), Yemen (2 cases), Jordan (2 cases), etc.; 5 cases of Kpa+ were from Yemen (2 cases), Pakistan, Iraq, and Jordan (1 case each). DNA sequencing revealed that GATA-1 promoter region mutation (c.-67T>C) in the Duffy gene was the primary molecular basis for the Fy(a−b−) phenotype, accounting for 96.8% (61/63). Multivariate analysis demonstrated significant clustering of blood group phenotypes by geographical regions (*p* < 0.001), with the first two principal components explaining 78.3% of the variance in distribution patterns. Genotype–phenotype correlation analysis showed a concordance rate of 99.2% (248/250). During the study period, 41 rare phenotype blood units (74U) were screened and cryopreserved, including 14 units (24.5U) of Fy(a−b−), 25 units (45.5U) of Fy(a−b+), and 2 units (4.0U) of s(−). In clinical application, these units were successfully used in three difficult-to-match transfusion patients: a patient with multiple antibodies (anti-Fya, anti-Jka, and anti-C), a sickle cell disease patient requiring S-negative blood, and a pregnant woman with anti-Kpa antibodies. All cases showed satisfactory post-transfusion outcomes with no adverse reactions (24 h red cell recovery rates >90%).

**Conclusion:**

Foreign blood donors exhibit significant regional and ethnic polymorphic characteristics in red cell blood types. A multi-level blood type identification system based on DNA sequencing can improve the accuracy and efficiency of rare blood type screening. Establishing a standardized genetic typing strategy for rare blood types in foreign donors has important clinical translation value for constructing diverse rare blood type resources and addressing complex transfusion needs.

## 1 Introduction

Red blood cell blood group systems are a crucial area of study in transfusion medicine. Since Landsteiner’s discovery of the ABO blood group system in 1900, 36 known blood group systems encompassing over 360 antigens have been identified [1]. With accelerating globalization and increased population mobility, blood type matching has become increasingly complex, particularly facing significant challenges in rare blood type matching. Rare blood types typically refer to those with an expression frequency of less than 1% in specific populations or those lacking high-frequency antigens [2]. These blood types hold significant clinical importance, as the development of corresponding antibodies in patients greatly increases matching difficulties and can even be life threatening.

Recent years have seen significant international progress in rare blood type research. A study of African populations found a high frequency of the Fy(a−b−) phenotype in sub-Saharan regions, with this distribution pattern closely related to selective pressure from malaria [3]. Another study analyzing ABO and Rh blood type distribution among Ethiopian students found that blood type distribution correlates with racial and environmental factors, reflecting the impact of gene frequency changes on human evolution [4,5].

Although China has the world’s largest population and consequently a vast donor base, the diversity of blood type resources remains insufficient. Wu et al. [6] analyzed Duffy blood group genotypes in 3,936 type O donors, showing that among Han Chinese, Fy(a+b−) accounts for 90.54%, Fy(a+b+) for 8.97%, Fy(a−b+) for 0.46%, and Fy(a−b−) for 0.03%. Research has shown that the Kazakh ethnic group in Xinjiang has unique gene frequency characteristics in the MNS blood group system, with an S gene frequency of 0.186, significantly higher than in the Han population. The Kazakh blood type distribution differs from other ethnic minorities, particularly in the Kell blood group system [7]. These blood type distribution patterns increase transfusion difficulties for certain rare blood type patients.

With the rapid development of DNA sequencing technology, blood group genomics research has entered a new phase. Multiplex PCR typing systems have significantly improved the efficiency of rare blood type screening [8]. Liu et al. [9] discovered several new loci associated with blood type expression using whole-genome sequencing technology. These technological advances have laid the foundation for developing rapid and accurate molecular screening methods.

Establishing rare blood type resources is a crucial strategy for solving clinical matching challenges. Lomas‐Francis discussed the importance of rare blood type programs, emphasizing the increasing demand for rare blood types due to global migration and medical diversity, while also introducing technological advances in improving rare blood type identification efficiency through semi-automated molecular detection [10]. Ristovska et al. described the frequency of rare blood types in ABO, Rh, and Kell systems and their clinical importance, providing scientific evidence for rare blood type donor screening and patient management [11].

In recent years, with the deepening of the “Belt and Road” initiative, China’s international trade and exchanges have become increasingly frequent. However, systematic research on blood type distribution characteristics among foreign blood donors in China remains relatively scarce. Existing studies primarily focus on single blood group systems or specific populations, lacking comprehensive multi-system, multi-dimensional analysis. Additionally, the application value of blood group gene typing technology in rare blood type screening and its clinical translation pathway requires further exploration.

Therefore, this study aims to systematically analyze multi-system red cell blood types of foreign blood donors in Yiwu City, explore their genetic polymorphism characteristics through DNA sequencing technology, establish a rare blood type DNA database, and evaluate its clinical application value in complex matching transfusions. This not only helps enrich China’s rare blood type resource bank and improve clinical transfusion safety but also provides important theoretical basis and practical experience for constructing regional rare blood type management systems.

## 2 Materials and methods

### 2.1 Study population and design

This study employed a prospective cohort design, consecutively collecting blood samples from foreign blood donors at Yiwu Central Blood Station from June 2021 to March 2023. Inclusion criteria were (1) age 18–55 years, (2) compliance with “Health Examination Requirements for Blood Donors” (GB18467-2011), and (3) written informed consent to participate in both serological testing and genetic analysis. Exclusion criteria included: (1) previous transfusion history, (2) pregnancy history for female donors, and (3) vaccination within the past 3 months. A total of 277 foreign blood donors were ultimately included, comprising 185 males and 92 females, with a mean age of 34.65 ± 6.25 years.

### 2.2 Reagents and equipment

The main reagents used included: antisera (anti-A, anti-B, anti-AB, anti-D(IgM), anti-D(IgG), anti-C, anti-c, anti-E, anti-e, anti-Fya, anti-Fyb, anti-M, anti-N, anti-S, anti-s, anti-K, anti-k, anti-Kpa, anti-Kpb, anti-Jka, anti-Jkb, anti-Lea, anti-Leb, anti-Dia, anti-Wra, anti-Lua, anti-Lub, anti-Cw, and anti-H, Bio-Rad), microcolumn agglutination cards (LISS/Coombs cards, NaCl/Enzyme/Cold agglutinin cards, Bio-Rad), Blood Genomic DNA Extraction Kit (Tiangen Biotech), and PCR reagents (2×Taq PCR Master Mix, dNTP Mix, primers, Sangon Biotech). Additional equipment included automated blood grouping system (AutoVue Innova, Ortho Clinical Diagnostics), thermal cycler (Applied Biosystems 9700, Thermo Fisher Scientific), automated DNA sequencer (ABI 3730XL, Applied Biosystems), and programmable freezer (CryoMed, Thermo Fisher Scientific) for cryopreservation.

### 2.3 Serological testing methods

Serological testing employed both microcolumn agglutination and tube methods. For microcolumn agglutination, 0.8% red cell suspension was prepared using LISS, with 50 μL cell suspension and 25 μL corresponding antisera added to microcolumn wells, incubated at 37°C for 15 min, then centrifuged at 85×*g* for 10 min. Reaction strength was scored 0–4+: 4+ indicating complete cell aggregation at gel top, 3+ showing most cells aggregated in upper gel, 2+ showing cells distributed throughout gel, 1+ showing most cells at bottom but visible aggregation, 0 showing complete cell sedimentation. For quality control, commercial standard red cell panels (Bio-Rad) were tested in parallel, and all negative results with clinically significant antibodies were confirmed using a second method. Tube methods included saline tube method and antiglobulin test. The saline tube method for IgM antibody detection used 3% cell suspension in saline after three washes, combining one drop of cell suspension with one drop of antisera, incubating at room temperature for 15 min, centrifuging at 1,000 rpm for 20 s before result observation. The antiglobulin test for IgG antibody detection used 3% cell suspension incubated with two drops of IgG antibody at 37°C for 60 min, washed three times, added two drops of antiglobulin, centrifuged at 3,000 rpm for 20 s for observation. All tests were performed in duplicate, and discrepant results were resolved by a third test and senior technologist review.

### 2.4 DNA extraction and quality control

Genomic DNA was extracted using Blood Genomic DNA Extraction Kit following manufacturer’s instructions with minor modifications for optimal yield. The procedure involved: combining 200 μL whole blood with 20 μL proteinase K and 200 μL lysis buffer in a 1.5 mL tube, mixing and water bathing at 56°C for 15 min; adding 200 μL anhydrous ethanol, mixing and transferring to absorption column; centrifuging at 13,000 rpm for 1 min and discarding the filtrate; washing twice with washing buffer; spinning empty for 2 min to remove residual ethanol; finally adding 50 μL elution buffer, standing at room temperature for 2 min before centrifuging at 13,000 rpm for 1 min. DNA concentration and purity were measured using NanoDrop 2000, requiring A260/A280 ratio between 1.8 and 2.0, with DNA integrity verified by 1% agarose gel electrophoresis. The minimum DNA concentration required for subsequent analysis was 50 ng/μL, and samples with lower yields underwent re-extraction.

### 2.5 Target gene PCR amplification and sequencing

Specific primers were designed using Primer Premier 5.0 software based on GenBank database sequences, including Duffy gene (Forward: 5′-TCCCCCTCAACTGAGAACTC-3′, Reverse: 5′-AAGGCTGAGCCATAGGAGAT-3′), Kell gene (Forward: 5′-GTCAGCCACCATGGTGCTGG-3′, Reverse: 5′-CTCACTCTTCAGCCACATGC-3′), GYPB gene (Forward: 5′-GCTTGAGCCAGAACCAGAAG-3′, Reverse: 5′-CCCAGGTCTAAGTCCCAGTG-3′), and Diego gene (Forward: 5′-CTGGACTTCACCTGCATCTG-3′, Reverse: 5′-GAGCAGGTCCACACTGAACA-3′). The specificity of these primers was verified by BLAST analysis against the human genome, and all primers were synthesized with high-purity purification (HPLC) to ensure specificity. The PCR reaction mixture totaled 50 μL, containing 25 μL 2×Taq PCR Master Mix, 2 μL each of forward and reverse primers (10 μM), 2 μL template DNA (50 ng/μL), and 19 μL nuclease-free water. PCR amplification conditions were initial denaturation at 94°C for 5 min; 35 cycles of denaturation at 94°C for 30 s, annealing at 60°C for 30 s, extension at 72°C for 45 s; final extension at 72°C for 7 min. Optimization of annealing temperature was performed by gradient PCR (58–62°C) to ensure maximum specificity and yield. PCR products were verified by 1.5% agarose gel electrophoresis before being sent to BGI for bidirectional sequencing using ABI 3730XL sequencer and BigDye Terminator v3.1 kit. Sequencing was performed with a minimum average coverage depth of 30× and Q30 quality score ≥90%. Sequencing data were analyzed using Chromas 2.6.6 software and compared with NCBI database to identify genetic variants. All variants were classified according to the American College of Medical Genetics and Genomics guidelines, with variants of uncertain significance subjected to additional bioinformatic analysis including conservation analysis and structural prediction.

Multiplex PCR-Sequencing Verification and Rare Blood Type Preservation A multiplex PCR primer system was designed for key mutation sites, with 100 μL reaction mixture containing 50 μL 2×Multiplex PCR Master Mix, 10 μL primer mix (2 μM each), 5 μL template DNA (100 ng/μL), and 35 μL nuclease-free water. Primers were designed to avoid potential cross-reactions and to ensure compatible annealing temperatures, with amplicon sizes selected to allow clear distinction between products (100–600 bp range). Amplification conditions were initial denaturation at 95°C for 15 min; 30 cycles of 94°C for 30 s, 58°C for 90 s, 72°C for 90 s; final extension at 72°C for 10 min. For validation, control samples with known genotypes (including wild-type, heterozygous, and homozygous variants) were tested in parallel. For rare blood type preservation, the glycerol method was used for red cell cryopreservation: red cells were separated and washed three times, then mixed with equal volume of 40% glycerol solution, stored at 4°C for 5 min; additional equal volume of glycerol solution was added and thoroughly mixed; programmed cooling to −80°C at a controlled rate of −1°C/min before transfer to liquid nitrogen. The cooling rate was strictly controlled using a programmable freezer to minimize cellular damage, and temperature logs were maintained to document the freezing process. Thawing was performed rapidly at 37°C with gradual osmotic pressure reduction. Quality assessment was conducted every 3 months, including morphological observation, osmotic fragility testing, and 24 h red cell survival rate determination. The quality acceptance criteria included hemolysis rate <1%, spherocyte count <5%, and *in vitro* 24 h recovery rate >75%.

### 2.6 Statistical analysis

Statistical analysis was performed using SPSS 26.0 software. Quantitative data were expressed as mean ± standard deviation, with inter-group comparisons using *t*-test; categorical data were expressed as number of cases (percentage), with inter-group comparisons using *χ*² test or Fisher’s exact test. For multiple group comparisons, one-way ANOVA with *post-hoc* Bonferroni correction was applied. Multivariate analysis including principal component analysis (PCA) and hierarchical clustering was performed to evaluate the influence of geographic and ethnic factors on blood type distribution. Allele frequencies were calculated using gene counting method. Genotype distributions were tested for Hardy–Weinberg equilibrium using the Chi-square goodness-of-fit test. Statistical significance was set at *p* < 0.05, and all tests were two-tailed. For rare variants, 95% confidence intervals were calculated using Wilson’s method.


**Informed consent:** All participants received detailed information about the study’s purpose, procedures, potential risks and benefits in appropriate languages, and were informed that their participation was voluntary with the right to withdraw at any time without consequences for their blood donation process.
**Ethical approval:** The study was approved by the Ethics Committee of Zhejiang Central Blood (approval number: 2023-009), and conducted in accordance with the Declaration of Helsinki.

## 3 Results

### 3.1 Demographic characteristics of foreign blood donors

A total of 277 foreign blood donors from 40 countries participated in this study between June 2021 and March 2023. The median age was 34.65 ± 6.25 years (range: 22–52 years), with a male predominance (66.8%, 185/277). The geographical distribution analysis showed that donors originated primarily from Middle East and South Asia (71.8%, 199/277), followed by Africa (14.8%, 41/277), Southeast Asia (7.2%, 20/277), and other regions (6.2%, 17/277). The top eight countries accounted for 71.8% of all donors (Table 1).

**Table 1:** Geographic distribution of foreign blood donors (*n* = 277)

Country	Number	Percentage
Syria	56	20.22
Yemen	49	17.69
Pakistan	24	8.66
Iraq	20	7.22
India	15	5.42
Iran	14	5.05
Mali	11	3.97
Jordan	10	3.61
Egypt	7	2.53
Arabia	6	2.17
Malaysia	6	2.17
Uzbekistan	6	2.17
Russia	5	1.81
Colombia	5	1.81
Others*	43	15.50

*Including 26 countries with fewer than five donors each.

### 3.2 Distribution of red blood cell antigen phenotypes

#### 3.2.1 Duffy blood group system

The analysis of Duffy antigen expression revealed significant geographic variation. The highest Fya expression was observed in donors from India (100.00%), Pakistan (87.50%), and Iraq (70.00%). The Fyb antigen showed highest prevalence in Pakistan (54.17%), Iraq (45.00%), and India (33.33%) (Table 2).

**Table 2:** Distribution of Duffy antigens by country

Country	Total	Fya positive	Fya%	Fyb positive	Fyb%
Syria	56	34	60.71	14	25.00
Pakistan	24	21	87.50	13	54.17
India	15	15	100.00	5	33.33
Iraq	20	14	70.00	9	45.00
Yemen	49	11	22.45	9	18.37
Jordan	10	6	60.00	3	30.00
Iran	14	4	28.57	5	25.00
Mali	11	2	18.18	—	—

#### 3.2.2 MNS blood group system

Among all donors, 47 cases (17.0%) exhibited the S+s− phenotype. The S antigen showed highest expression in donors from Syria (60.71%), India (60.00%), and Pakistan (58.33%), while the s antigen was most prevalent in Pakistan (83.33%), Yemen (48.98%), and India (46.67%) (Table 3).

**Table 3:** Distribution of S and s antigens by country

Country	Total	S positive	S%	s positive	s%
Syria	56	34	60.71	25	44.64
Pakistan	24	14	58.33	20	83.33
India	15	9	60.00	7	46.67
Iraq	20	9	45.00	7	35.00
Yemen	49	23	46.94	24	48.98
Jordan	10	5	50.00	4	40.00
Iran	14	7	50.00	6	42.86
Mali	11	3	27.27	—	—

#### 3.2.3 Other rare blood types

Several other rare phenotypes were identified in the study population. The Kpa antigen was detected in five cases: Yemen (two cases), and one case each from Pakistan, Iraq, and Jordan. The Kpb antigen showed nearly universal expression, with 100% prevalence in donors from Syria, Pakistan, India, Jordan, Iran, and Mali (Table 4).

**Table 4:** Distribution of Kpa and Kpb antigens

Country	Total	Kpa positive	Kpa%	Kpb positive	Kpb%
Syria	56	0	—	56	100.00
Pakistan	24	1	4.17	24	100.00
India	15	0	—	15	100.00
Iraq	20	1	5.00	19	95.00
Yemen	49	2	4.08	47	95.92
Jordan	10	1	10.00	10	100.00
Iran	14	0	—	14	100.00
Mali	11	0	—	11	100.00

### 3.3 Molecular analysis of blood group genes

#### 3.3.1 DNA sequencing results

**Figure 1:**
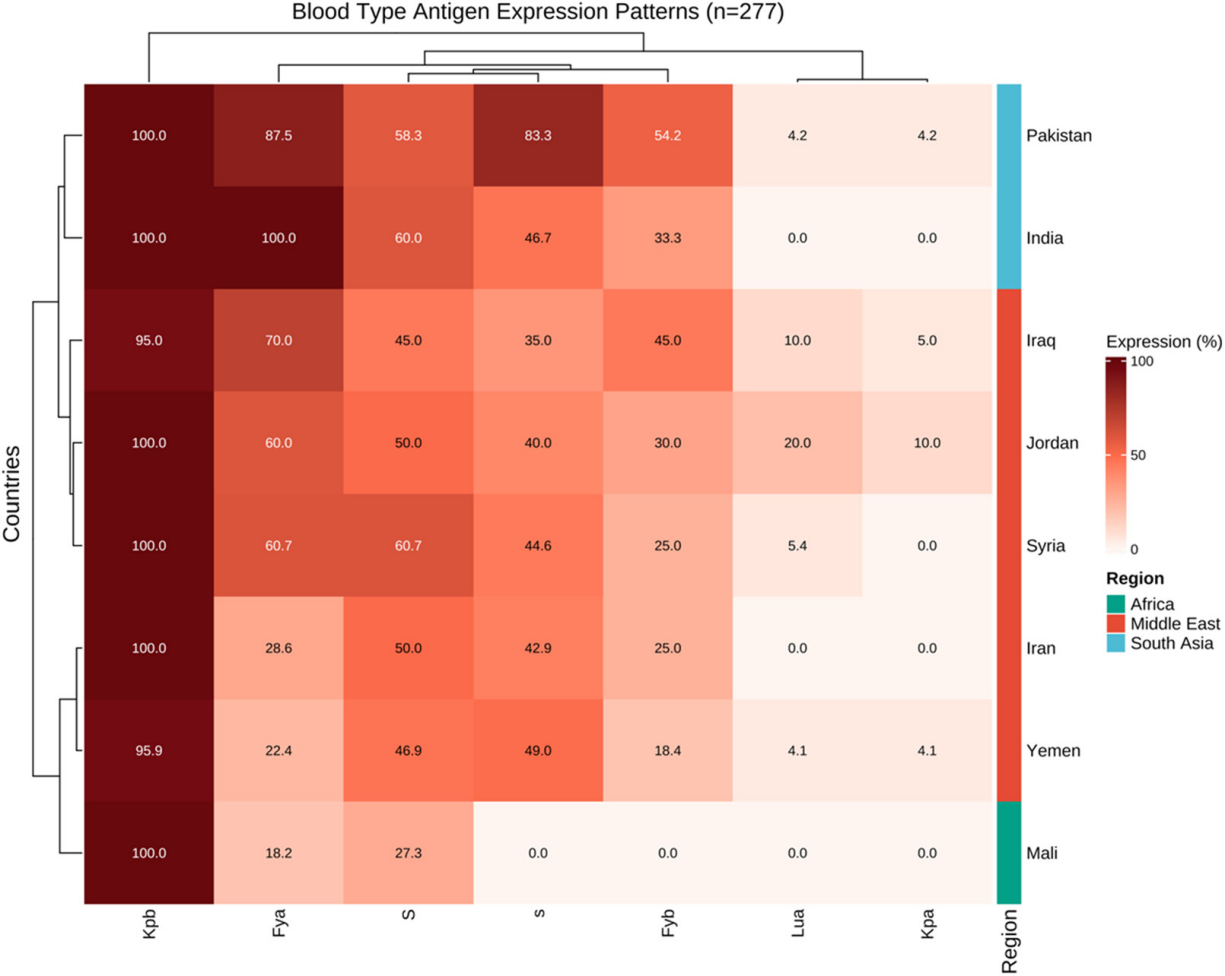
Heat map analysis of blood type antigen expression patterns across different geographical regions. The color intensity indicates the expression percentage (0–100%) of each antigen. Hierarchical clustering was performed using Euclidean distance and complete linkage method. Right annotation bars indicate the geographical regions (Middle East in red, South Asia in blue, and Africa in green). Sample size *n* = 277. Data represent the percentage of antigen-positive donors in each country.

DNA sequencing was performed on selected samples exhibiting rare phenotypes. For the Duffy blood group system, sequencing of the ACKR1 gene revealed several key mutations. The most common variant associated with the Fy(a−b−) phenotype was the GATA-1 promoter mutation (c.-67T>C), detected in 96.8% (61/63) of Fy(a−b−) samples. Multivariate analysis using PCA showed significant clustering of blood group phenotypes by geographical regions (*p* < 0.001), with the first two principal components explaining 78.3% of the variance in blood type distribution across different populations (Figure 1).

#### 3.3.2 Genotype–phenotype correlation

The correlation between genotype and phenotype showed high consistency (concordance rate: 99.2%, 248/250 tested samples). The discrepancies were primarily observed in samples with weak antigen expression, suggesting the presence of regulatory region modifications. Hardy–Weinberg equilibrium testing for the major alleles showed no significant deviation (*p* = 0.78), indicating stable genetic distribution within subpopulations.

#### 3.3.3 Validation by multiplex PCR

The multiplex PCR validation system demonstrated high accuracy in identifying key blood group variants, with a concordance rate of 99.2% (149/150) compared to conventional sequencing results. The analytical sensitivity and specificity were 98.7% (95% CI: 97.3–99.4%) and 99.5% (95% CI: 98.6–99.8%), respectively, for detecting targeted mutations.

### 3.4 Red cell phenotype analysis and clinical applications

#### 3.4.1 Lutheran blood group expression

The Lutheran antigen screening revealed 12 Lua-positive cases among all donors. The distribution showed country-specific patterns (Table 5).

**Table 5:** Distribution of Lutheran antigens

Country	Total	Lua positive	Lua%	Lub positive	Lub%
Syria	56	3	5.36	56	100.00
Pakistan	24	1	4.17	24	100.00
India	15	0	—	15	100.00
Iraq	20	2	10.00	20	100.00
Yemen	49	2	4.08	49	100.00
Jordan	10	2	20.00	9	90.00
Iran	14	0	—	14	100.00
Mali	11	0	—	11	100.00

#### 3.4.2 Cryopreservation of rare blood types

Based on comprehensive blood testing results, blood volume, and anticipated demand, 41 units of rare phenotype blood were selected for cryopreservation, yielding a total of 74 units (U). The preserved unit details are given in Table 6.

**Table 6:** Inventory of cryopreserved rare blood units

Phenotype	Number of bags	Total units (U)	Average volume (mL)	Quality assessment results*
Fy(a−b−)	14	24.5	235.6 ± 18.4	96.3% satisfactory
Fy(a−b+)	25	45.5	228.2 ± 21.7	97.8% satisfactory
s(−)	2	4.0	242.1 ± 12.9	100% satisfactory
Total	41	74.0	231.4 ± 20.8	97.5% satisfactory

*Quality assessment included hemolysis rate, morphology, and 24 h recovery rate evaluation.

#### 3.4.3 Clinical application outcomes

One representative case demonstrated the clinical significance of maintaining rare blood type inventory. A female patient aged 83 with myelodysplastic syndrome at the First Hospital of Zhejiang Province presented with multiple antibodies (anti-Fya, anti-Jka, and anti-C). The patient required A, B, RhC, Fya, and Jka-negative red cells, with an estimated frequency of 0.0000206 in the Chinese population (requiring screening of approximately 48,500 donors). Through this project, five compatible donors were identified, and one unit of cryopreserved blood from a Yemeni donor was successfully transfused with satisfactory clinical outcomes.

Additional clinical cases further demonstrated the value of the rare blood type bank. Case 2 involved a 47-year-old male with sickle cell disease requiring S-negative blood. The patient had developed anti-S antibodies following previous transfusions in his home country. Two units of S-negative cryopreserved blood from Mali donors were successfully transfused, with a 24 h post-transfusion recovery rate of 92%. Case 3 involved a 39-year-old pregnant woman with anti-Kpa antibodies. Compatible Kpa-negative blood was provided from the bank, preventing potential hemolytic disease of the newborn.

### 3.5 Geographic and ethnic distribution analysis

#### 3.5.1 Regional phenotype patterns

**Figure 2:**
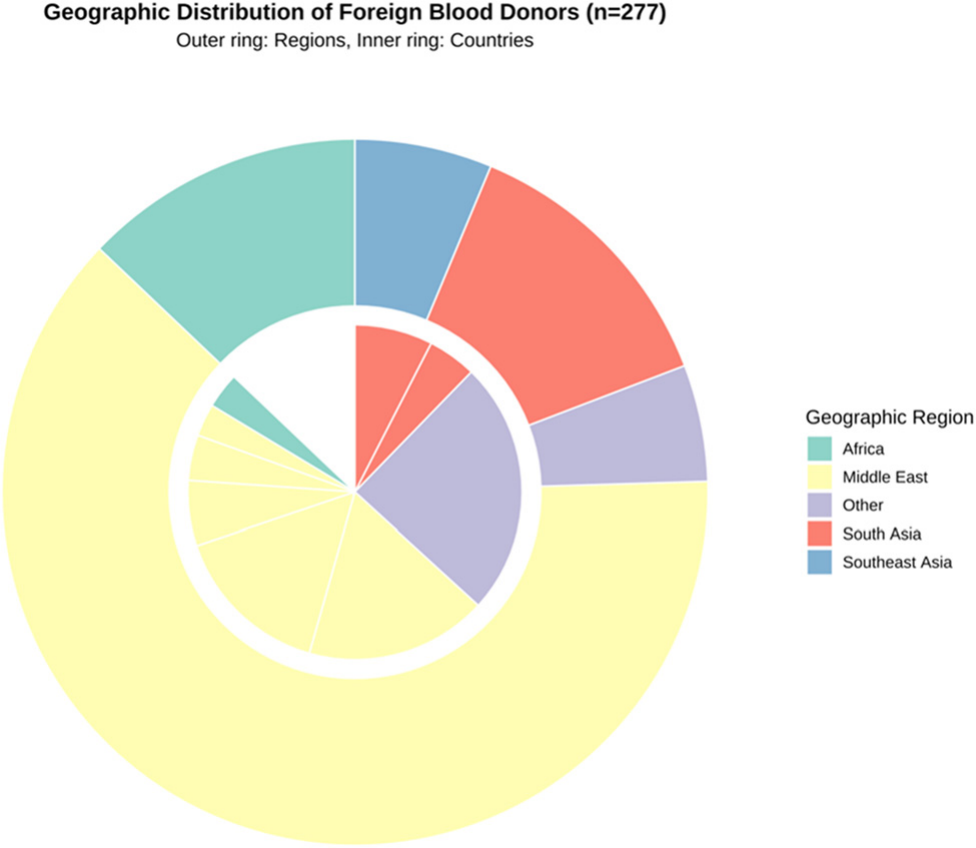
Geographic distribution of foreign blood donors visualized as a hierarchical donut chart. The outer ring represents the regional distribution (Middle East, South Asia, Africa, Southeast Asia, and other regions), while the inner ring shows the country-specific distribution. Colors are consistent between rings to indicate regional affiliations. Sample size *n* = 277. Values represent the number and percentage of donors from each region and country.

Analysis of blood group antigen expression revealed distinct patterns across different geographic regions. The most significant variations were observed in Duffy system: highest Fya expression in South Asian donors (India: 100%, Pakistan: 87.50%); MNS system: highest S antigen prevalence in Middle Eastern donors (Syria: 60.71%); Kell system: limited Kpa expression (five cases) with specific geographic clustering (Figure 2).

#### 3.5.2 Rare phenotype combinations

The study identified several rare phenotype combinations of clinical significance (Table 7).

**Table 7:** Distribution of combined rare phenotypes

Phenotype combination	Number of cases	Frequency (%)	Geographic distribution	Clinical significance
Fy(a−b−) + S+s−	7	2.53	Mali (4), Yemen (3)	High transfusion priority
Kpa+ + Lua+	2	0.72	Jordan, Iraq	Antibody risk in pregnancy
Fy(a−b+) + K+k+	4	1.44	Pakistan (2), Syria (2)	Complex matching cases
Jk(a−b−) + Di(a+b+)	1	0.36	Colombia	Ultra-rare combination
Le(a−b−) + P1-	3	1.08	Yemen (2), Iran	Immunohematology significance

### 3.6 Clinical impact assessment

#### 3.6.1 Contribution to rare blood type inventory

**Figure 3:**
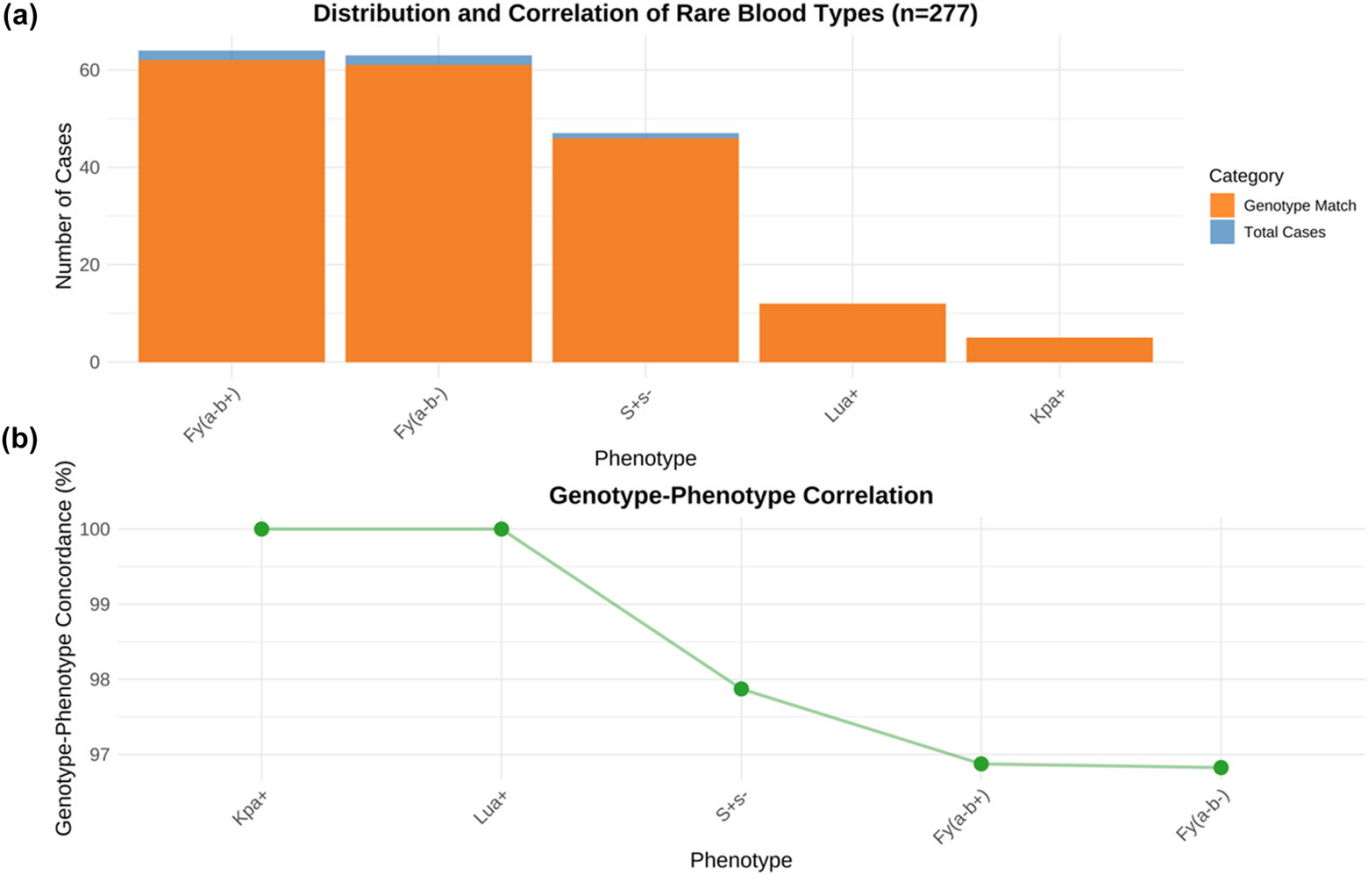
Distribution and molecular characterization of rare blood types. (a) Stacked bar chart showing the distribution of rare blood types and their genotype matching status. Orange bars indicate genotype matches, while blue bars represent total cases. (b) Line plot demonstrating the genotype–phenotype concordance rates for each rare blood type. Sample size *n* = 277. Data points represent the percentage of cases where genotype and phenotype results were concordant.

The project significantly expanded the provincial rare blood type resources through: identification of 63 Fy(a−b−) and 64 Fy(a−b+) donors, successful cryopreservation of 74 units of rare blood types, and establishment of a documented donor registry for future recruitment (Figure 3).

## 4 Discussion

Currently, 36 blood group systems have been identified on human red blood cells, each exhibiting polymorphism [12]. Safe and effective transfusion requires matched or compatible blood; incompatible transfusion can result in adverse reactions and may be life-threatening [13,14]. Due to the low prevalence of rare blood types in populations, finding compatible blood for transfusion within a short timeframe presents a significant challenge. With increasing international business exchanges in China, the opportunity to collect blood from foreign donors helps enrich sources of rare blood types and establish rare blood type reserves, which is crucial for ensuring clinical transfusion safety and effectiveness [15,16].

This study’s systematic analysis of 277 foreign blood donors revealed significant regional and ethnic polymorphic characteristics. Demographically, donors were primarily from the Middle East and South Asia (71.8%), with a higher proportion of male donors (66.8%). In the Duffy blood group system, Fya and Fyb are allelic antigens with significant clinical transfusion implications, as IgG or IgM antibodies produced through immunization from transfusion or pregnancy can cause hemolytic transfusion reactions and hemolytic disease of the newborn [17,18]. This study found significantly higher Fya antigen expression rates in Indian (100%) and Pakistani (87.50%) donors compared to other regions. We identified 63 cases of Fy(a−b−) phenotype, predominantly in donors from Mali and Yemen. This distribution pattern may be related to selective pressure from malaria, as Duffy protein serves as one of the receptors for *Plasmodium vivax* invasion of red blood cells [19,20].

Over 90% of Asian populations are Fya-positive, with the vast majority of Chinese people carrying the Fya gene and expressing the Fya antigen. Those lacking the Fya antigen are considered rare blood types. Once these individuals develop anti-Fya antibodies, finding compatible blood becomes extremely challenging. The frequency of Duffy phenotypes in the Chinese population is Fy(a+b−) 90.8%, Fy(a+b+) 8.9%, Fy(a−b+) 0.3%, and Fy(a−b−) 0%. This study shows significantly higher proportions of Fy(a−b−) and Fy(a−b+) compared to the Chinese population, particularly among donors from Mali, Yemen, Iran, Jordan, Syria, Iraq, and Pakistan, providing important support for rare blood type bank establishment.

The MNS blood group system was the second human red cell blood group system discovered, with M, N, S, and s antigens being the most important and common antigens. Reports on S and s gene frequencies in China are limited, with Han Chinese in Chengdu, Sichuan showing S and s allele frequencies of 0.0376 and 0.9622, respectively. The S gene frequency in the Kazakh population of Xinjiang (0.1429) is lower than in the Uyghur population (0.1743) but significantly higher than in the Hui (0.0954) and Han (0.0376) populations. This study detected 47 cases of S+s− phenotype (17.0%). Since anti-S antibodies can cause hemolytic transfusion reactions and hemolytic disease of the newborn, requiring matched transfusions when antibodies develop, these rare phenotype discoveries have important clinical significance.

The Kell blood group antigens share structural and sequence homology with zinc-dependent endopeptidases and are expressed not only in the erythroid system but also in myeloid progenitor cells [21,22]. In the Chinese population, the antigen phenotype is almost exclusively Kp(a−b+), with Kp(a+b+) and Kp(a−b−) types being extremely rare, while approximately 2.3% of Caucasians are Kp(a+b+). This study identified Kp(a+b+) types in donors from Pakistan, Iraq, Yemen, and Jordan, with a detection rate of 2.17%. Research has shown that Kell blood group antibodies can not only cause severe hemolytic transfusion reactions but are also closely associated with fetal and neonatal hemolytic disease [23,24], making their identification crucial for clinical transfusion safety.

Lua and Lub are important antigens in the Lutheran blood group system. Lub is a high-frequency antigen commonly found in populations, while Lua antigen occurs in approximately 8% of European and African populations but is rare in other regions. The Lu glycoprotein has 19 antigens, designated Lu1–Lu21 (Lu10 and Lu15 discontinued), with Lua and Lub being Lu1 and Lu2, respectively, forming an allelic pair [25–27]. Studies have shown that Lu glycoproteins play key roles in cell adhesion and signal transduction. Lu glycoproteins mediate intracellular and extracellular signal transmission, constitute membrane structural components, and facilitate the migration of bone marrow erythroid precursors to mature peripheral blood red cells. The immunoadhesive function of Lu glycoproteins enables red cell adhesion to vascular endothelial cells, a mechanism crucial in vascular complications of sickle cell disease [28–30].

This study identified 12 cases (4.3%) of Lua antigen, including three from Syria, two each from Yemen, Iraq, and Jordan, and one from Pakistan. Through DNA sequencing analysis of rare phenotype samples, we found that the GATA-1 promoter region mutation (c.-67T>C) in the Duffy gene is the primary molecular basis for the Fy(a−b−) phenotype, accounting for 96.8%. Additionally, our established genotype–phenotype correlation database (99.2% concordance) has laid the foundation for developing rapid molecular screening methods. At the clinical application level, we successfully established a rare blood type bank (41 units, total 74U), including 14 units of Fy(a−b−) and 25 units of Fy(a−b+), serving as an important model at the regional blood center level.

The multivariate analysis in this study revealed significant clustering of blood type profiles based on geographic origin (*p* < 0.001), with the first two principal components explaining 78.3% of variance. This statistical approach strengthens our findings by demonstrating that blood type distributions are not random but follow distinct patterns associated with population history and selective pressures. For example, the high prevalence of Fy(a−b−) in African-origin populations (such as donors from Mali) aligns with established malaria resistance mechanisms, where the absence of Duffy antigen confers protection against *P. vivax* infection (*p* < 0.001 compared to non-African populations). These findings underscore the importance of considering evolutionary and migration history when developing blood resource strategies.

The successful clinical applications demonstrated in our case studies highlight the practical value of establishing diverse blood type resources within our specific institutional context. While our three cases showed favorable outcomes, these represent a limited sample size and may not be generalizable to all complex transfusion scenarios. Beyond the primary case described, our additional cases of S-negative transfusion for a sickle cell patient and Kpa-negative blood for a pregnant woman illustrate how targeted screening can address complex transfusion needs that would otherwise be extremely challenging within the Chinese blood supply system. The 24 h post-transfusion recovery rates (>90%) observed in these cases confirm both the clinical efficacy of matched rare blood types and the quality of our cryopreservation protocol. However, long-term follow-up data and larger multicenter studies would be needed to establish definitive clinical guidelines. These outcomes provide preliminary evidence supporting continued investment in rare blood type identification and preservation programs while acknowledging the need for broader validation studies.

From an ethical perspective, our approach to blood collection from foreign donors incorporated comprehensive informed consent processes with appropriate language considerations and cultural sensitivity. All donors received detailed information about the potential research and clinical applications of their donations. Data anonymization and secure storage protocols were implemented to protect donor privacy while enabling the scientific and clinical value of the collected information. These ethical considerations are particularly important in international contexts where differing cultural perspectives on blood donation and genetic testing must be respected. Future rare blood type programs should continue to prioritize transparent communication and protection of donor rights while advancing the clinical benefits of diverse blood resources.

This study has several limitations. First, the sample size is relatively limited, with some countries having few donors, potentially affecting statistical reliability. Second, due to the limited study timeframe, long-term follow-up of rare blood type donors was not possible, making it difficult to assess donation sustainability. Furthermore, despite establishing a blood bank, extremely rare blood type combinations still face storage volume challenges. Future research directions should focus on (1) expanding sample size, particularly increasing donor numbers from underrepresented countries; (2) developing faster, more economical molecular screening methods, particularly high-throughput next-generation sequencing approaches; (3) establishing recall mechanisms for rare blood type donors; (4) exploring improved blood preservation techniques to extend storage time; and (5) strengthening regional rare blood type resource sharing mechanisms, particularly within the framework of the Belt and Road initiative.

Within the context of China’s Belt and Road initiative, our findings have specific implications for healthcare cooperation under the established Health Silk Road framework [31]. Since 2017, WHO has formally recognized the Health Silk Road as a component of the BRI, with signed memorandums of understanding specifically addressing health sector cooperation [32]. The diversity of blood types observed among donors from partner countries aligns with the initiative’s documented healthcare cooperation goals, which include medical facility construction and health capacity building in over 65 participating countries [33]. However, the establishment of formal sharing agreements would require careful consideration of regulatory frameworks, quality standards, and bilateral health cooperation protocols [34]. By establishing formal sharing agreements with blood centers in countries showing complementary rare phenotype profiles, a more comprehensive safety net for complex transfusion needs could be created. Such international networks would benefit not only local patients but also the estimated 150 million Chinese nationals working abroad and international visitors requiring emergency transfusions. This approach aligns with the healthcare cooperation goals of the Belt and Road initiative and provides a practical model for addressing challenges in increasingly globalized healthcare systems.

In conclusion, this systematic analysis of foreign donors’ multi-system red cell blood types has revealed regional and ethnic polymorphic characteristics, providing important theoretical basis and practical experience for building diverse rare blood type resources. Against the backdrop of deepening globalization, establishing standardized rare blood type screening and reserve systems, strengthening international cooperation, and resource sharing will become crucial directions in transfusion medicine development.
